# A Primer on Quality Assurance and Performance Improvement for Interprofessional Chronic Kidney Disease Care: A Path to Joint Commission Certification

**DOI:** 10.3390/pharmacy7030083

**Published:** 2019-07-03

**Authors:** Linda Awdishu, Teri Moore, Michelle Morrison, Christy Turner, Danuta Trzebinska

**Affiliations:** 1 School of Pharmacy and Pharmaceutical Sciences, University of California, San Diego, CA 92093, USA; 2 Nephrology Department, School of Medicine, University of California, San Diego, CA 92093, USA

**Keywords:** chronic kidney disease, interprofessional care, quality assurance

## Abstract

Interprofessional care for chronic kidney disease facilitates the delivery of high quality, comprehensive care to a complex, at-risk population. Interprofessional care is resource intensive and requires a value proposition. Joint Commission certification is a voluntary process that improves patient outcomes, provides external validity to hospital administration and enhances visibility to patients and referring providers. This is a single-center, retrospective study describing quality assurance and performance improvement in chronic kidney disease, Joint Commission certification and quality outcomes. A total of 440 patients were included in the analysis. Thirteen quality indicators consisting of clinical and process of care indicators were developed and measured for a period of two years from 2009–2017. Significant improvements or at least persistently high performance were noted for key quality indicators such as blood pressure control (85%), estimation of cardiovascular risk (100%), measurement of hemoglobin A1c (98%), vaccination (93%), referrals for vascular access and transplantation (100%), placement of permanent dialysis access (61%), discussion of advanced directives (94%), online patient education (71%) and completion of office visit documentation (100%). High patient satisfaction scores (94–96%) are consistent with excellent quality of care provided.

## 1. Introduction

Interprofessional (IP) care for chronic kidney disease (CKD) facilitates the delivery of comprehensive care to a complex, at-risk population. Evidence based strategies for slowing progression of CKD are well described but not consistently applied to the individual patient. Interprofessional teams focus on implementing evidenced based care to slow down the progression of CKD, educating patients about their disease and streamlining the transition to end stage renal disease (ESRD). This comprehensive care has been shown to reduce hospitalizations [1,2,3,4,5,6] lower mortality [1,3,4,7,8,9], slow the progression of CKD [1,2,3,10,11] and prepare patients for transitions in care [4,7,8]. Despite these benefits, IP CKD programs are difficult to implement because of resources. Advocates of IP care should provide evidence of value to justify the additional cost since CKD consumes a disproportionate share of healthcare funding globally [12,13]. The Joint Commission (TJC) certification is a voluntary process that can improve patient outcomes, provides external validity to hospital administration and enhances visibility to patients and referring providers. In this paper, we describe the development of an IP CKD program and the pathway to TJC disease specific certification in CKD care.

## 2. Materials and Methods

This is a retrospective, single center study of all adult patients receiving care in the IP CKD Program from July 2011–2016. We included all adult patients with CKD receiving IP care. Patients with less than 3 months of follow up in the program were excluded from the analysis. Clinical data including patient demographics, comorbidities, laboratory results, vital signs measured during clinic visits, medications, process of care measures as well as outcomes of dialysis initiation, transplantation or death were extracted from the electronic health record (EHR). To analyze patient demographics and performance measures for each recertification cycle, we used descriptive statistics including mean and standard deviation (SD) or median and range for continuous data when appropriate, frequency counts and percentage for categorical data. Descriptive statistics were calculated using R version (3.4.2). The institutional review board (IRB) for human subjects approved this study with a waiver of consent.

### 2.1. Program Description

The pharmacist wrote the proposal for the IP CKD Program and obtained funding from the health system. This program was set up as its own cost center and the pharmacist funding is supported by the health system for direct patient care and administration of the CKD program. The nephrologist and pharmacist created the infrastructure for the program including a mission statement, clinic schedules, job descriptions, template notes and standard orders. Our institutional IP CKD Program opened in 2007 and provides comprehensive care to patients with CKD stage 2 through 5. Patients are referred for IP care by a nephrologist or through direct referral from other disciplines. The program does not have specific referring criteria; rather any patient who the referring physician feels would benefit from IP CKD care is eligible. The program consists of two half-day clinics, which operate in the same physical space as other nephrology clinics. Patients are seen every 1 to 6 months depending on disease severity. Visits are approximately 90 and 45 min for new and return patients, respectively. The core IP team consists of a nephrologist/medical director, pharmacist/program administrator, nurse, dietitian, social worker and patient education coordinator. Interprofessional care is provided based on institutional CKD guidelines and documented in the EHR. Chronic kidney disease guidelines were developed using Kidney Disease Improving Global Outcomes (KDIGO) guidelines, Kidney Disease Outcomes Quality Initiative (KDOQI), Joint National Commission hypertension guidelines, American Diabetes Association diabetes care guidelines, American Heart Association guidelines as well primary and tertiary references [14,15,16,17,18,19,20,21,22,23,24,25,26,27,28,29,30,31,32,33,34,35,36,37,38,39,40,41,42,43,44,45,46,47,48,49,50,51,52,53,54,55,56,57,58,59,60,61,62,63,64,65,66,67,68,69]. These were approved by our institutional Pharmacy and Therapeutics Committee and updated annually.

Patients receive many services in addition to the traditional nephrology care such as cardiovascular risk assessment, dietary counseling on CKD diet, weight loss, vaccinations, smoking cessation, medication reconciliation and management, personalized medication schedules, assistance with insurance and transportation issues as well as assistance with transition of care to transplant, dialysis or hospice (Appendix A). Patient education is provided during each visit on an individual basis, through online educational videos (https://www.youtube.com/playlist?list=PLp5o_4MxOoYRJ_zYObvWzC-O0VE7wFqaG) and in a classroom setting. Educational topics include introduction to CKD, medications and CKD, diet, social support networks, renal replacement modalities and transplantation.

Each team member has defined roles and responsibilities to optimize patient care. All members of the IP team receive orientation to the program and institutional CKD guidelines. Team members are evaluated for competency initially and must demonstrate continuing competency and education annually. Competency is assessed verbally and through the demonstration of skills where appropriate. Team members answer questions about mini case scenarios to test their knowledge of CKD staging, overall goals of care based on CKD stage, laboratory parameters and foods high in phosphorus and potassium. Skills such as blood pressure measurement or administration of medications are evaluated by demonstration. Contemporary topics in CKD care are reviewed during a mandatory monthly journal club. The Medical Director and Program Administrator conduct annual performance reviews for all team members incorporating 360-degree feedback from team members.

### 2.2. Design of Quality Assessment and Performance Improvement Program (QAPI) and CKD Registry

The IP team determined appropriate quality indicators consisting of clinical, process and financial measures such as blood pressure (BP) control, prevalence of permanent vascular access at dialysis initiation, vaccination rates, patient education among others (Table 1). These measures were chosen on the basis of their importance for delaying CKD progression, streamlining transitions in care, improving patient experience and applicability to the majority of the program population. Each quality indicator was defined, baseline and targets established and strategies were developed to achieve target goals. For example, we defined BP control as the percentage of patients achieving the target BP per Joint National Committee guidelines. We established our baseline rate of control and set a target for improvement. Strategies to achieve the target included medical assistant education on performing a BP measurement, providing home BP monitors and logs to patients for home monitoring, patient education and nursing telephone follow up for patients with uncontrolled hypertension.

We developed a CKD registry in the EHR enabling the automated reporting of CKD outcomes. Data is electronically extracted and presented in a QAPI dashboard, which is reviewed on a monthly basis by the IP team and submitted to TJC. All outliers are reviewed in detail during the monthly team meeting and new strategies to achieve targets are developed on an as needed basis.

## 3. Results

A total of 440 patients currently receive care in the IP CKD program. The demographics are summarized in Table 2.

The mean age of the population is 64.2 ± 14.5 years, 55% are male and the majority are White with 24% Hispanic patients as the second largest ethnicity. The majority of patients are in CKD stage 3 (51%) followed by stage 4 (24%) and stage 5 (17%). Approximately half of the patients have diabetes and 92% have hypertension.

Prior to the first certification in 2010, we chose the following quality indicators: (1) blood pressure control (median systolic and diastolic blood pressure, percent of patients with SBP ≤ 130 mmHg, percent of patients with SBP ≤ 140 mmHg), (2) median hemoglobin, (3) screening for appropriate patient referrals to vascular surgery and transplant and (4) percent of patients with education about CKD within 3 months of clinic enrollment (Table 3).

We collected 6 months of data on those measures for the initial certification. The range of median systolic blood pressure was 127–137 mmHg, 36–44% of patients had SBP ≤ 130 mmHg, and 56–76% of patients had SBP ≤ 140 mmHg. Eighty-eight to 100% of patients had an appropriate referral to vascular surgery or transplantation. The percentage of patients who received in classroom CKD education within the first 3 months of joining the clinic steadily rose from 33% to 50%. We received Disease Specific Certification for CKD with no findings for improvement and were noted to be the first program in the United States with this designation. After receiving our certification, our surveyor invited us to present our program outcomes to the Quality Net Conference for the Centers for Medicare and Medicaid.

In the first recertification cycle 2011–2013, we chose the following indicators: (1) SBP ≤ 130 mmHg (2) pneumococcal vaccination rate (3) discussion of advanced directives (4) office visit cancellation rate (Table 4).

Tight control of SBP ≤ 130 mmHg was achieved in 47–58% of patients and SBP ≤ 140mmHg was obtained in 74–85% of patients. Pneumococcal vaccination included Prevnar 13 and Pneumovax 23 and the rate rapidly rose from 49% in the first quarter to 93% in the last quarter of the cycle, as did the percentage of patients who discussed advanced directives with our social worker, from 29% to 94% over a two-year period. We were not able to sustain a decrease in the rate of cancellation of office visits after an initial drop from 28% to 19%.

In the second recertification cycle 2013–2015, we chose the following quality indicators: (1) SBP ≤ 130 and 140 mmHg (2) percentage of patients starting hemodialysis (HD) with AVF or arteriovenous graft (AVG), (3) median days from referral to first appointment in CKD clinic and (4) percent of office notes closed in EHR within 48 h (Table 5).

In December 2013, the Joint National Commission released new guidelines recommending a blood pressure goal of ≤140 mmHg and we decided to stop tracking the SBP goal ≤130 mmHg. We achieved SBP control to ≤140 mmHg in 79–85% of patients. The percentage of patients starting dialysis with AVF or AVG varied from 25 to 100% in different quarters with overall average of 77%. The median wait time from referral to first CKD clinic appointment varied from 7 to 37 days. The percentage of note closure within 48 h improved from 45% to 100%.

In the third recertification cycle 2015–2017, the following quality indicators were chosen: (1) patient viewing of online education videos, (2) continuation of permanent dialysis access indicator, (3) ordering of hemoglobin A1c every 6 months for patients with diabetes and (4) estimation and documentation of atherosclerotic cardiovascular disease (ASCVD) risk (Table 6).

Fifteen patient education videos were created to improve treatment adherence, self-care, and clinical outcomes for CKD patients. CKD team members, including the nephrologist, pharmacist, dietitian, and social worker, each created several 5–15 min videos on specific topics related to CKD patient care that were peer reviewed by all team members. The videos were filmed in English at a production studio located on the main university campus. The entire process of video planning and filming took three months. After production of videos was complete, a CKD playlist of videos was created and published on YouTube (https://www.youtube.com/playlist?list=PLp5o_4MxOoYRJ_zYObvWzC-O0VE7wFqaG). A link to the videos was shared through e-mail, EHR patient messaging and posted on the CKD program website. A brochure advertising the videos was created and distributed to patients in clinic and through mail. For patients without computers, internet access, or mobile phones, a DVD was distributed in clinic or the video was played for the patient on clinic computers. Each patient was asked about video views during the office visit and this was documented in the EHR using smart fields for electronic data extraction. There was a steep and consistent increase in the percentage of patients viewing our online education videos from 0 to 71%. The 15 videos received a total of 284,808 views and the total number of views per videos ranged from 276 to 132,710 far surpassing our patient population. Videos with the highest views included content on: (1) symptoms of kidney disease (132,710 views), (2) stages of kidney disease (91,265 views), and (3) laboratory values of kidney disease (18,615 views).

For the other quality indicators, the percentage of permanent dialysis access at dialysis initiation was 0–100% (median 61%), and, the number of patients starting dialysis was low ranging from 0–3 per month. Hemoglobin A1c testing was high at baseline at 90% and remained consistently high with a range of 89–98%. ASCVD risk was not routinely documented in office visit notes at baseline. After implementation of an automated ASCVD risk estimate calculator in the EHR, we demonstrated an immediate increase in documentation to 82% in the first quarter of implementation and subsequent increase to 100% documentation. We received re-certification with no findings for improvement and positive feedback on the successful development of online education for patients with CKD.

Patient satisfaction was measured using The Consumer Assessment of Health Providers and Systems surveys administered by Press Ganey and collected for each certification and recertification cycle. From 2012 to present, the percentage of surveys where patients reported “yes, definitely” on a 3-point scale for their likelihood to recommend the program and the physician communication domain were approximately 94% and 96%, respectively.

## 4. Discussion

In this study, we demonstrated that IP care for CKD could be implemented and sustained over a long time period at an academic institution. The process of obtaining TJC certification is educational and rewarding. It provides opportunity to examine program performance and identify gaps in care. TJC certification ensures an ongoing process of quality measure development, implementation of interventions to achieve program goals and measurement of outcomes. In contrast to other regulatory agencies, TJC certification provides flexibility for programs to determine their own meaningful measures of performance. Over the past eight years, we have defined and measured 13 quality indicators for CKD care. Overall, we were able to improve performance on the majority of quality indicators or at least maintain the high performance. Most indicators were retired at the end of the recertification cycle. Some were considered critical to CKD care and were continued in additional cycles. 

### 4.1. Blood Pressure Control

Blood pressure control remained a performance measure for 3 cycles of recertification since it is essential to preventing CKD progression and we found opportunity for performance improvement. On average, blood pressure control was achieved in 81% of patients. When we targeted a more stringent goal (<130/80 mmHg), between 53–67% of our patients were able to achieve that target. Despite a drop in control with implementation of more stringent targets, we still achieved higher rates of control compared to the literature. Thanamayooran and colleagues demonstrated that 40% of patients achieved a target blood pressure of <130/80 mmHg when receiving IP CKD care [75]. Surveys of the general population have demonstrated that 13.2–37% of patients with CKD achieve a target blood pressure [76,77].

Achieving a target clinic blood pressure proved to be challenging. To improve blood pressure control, the entire IP team was engaged in numerous aspects. Our medical assistants have yearly competency evaluation on accurate blood pressure measurement and ensure elevated blood pressure measurements are repeated and recorded. The dietitian counsels on dietary sodium restriction and educates patients on how to read food labels. The pharmacist assesses medication adherence, adverse effects of antihypertensives and optimizes therapy. Nurses perform routine telephone follow up on home blood pressure measurements for patients whose clinic measurements are not at goal. The social worker assesses financial resources and addresses barriers to medication access and provides a free blood pressure monitor to patients in need. The physician reviews the team recommendations, summarizes a plan that optimizes the antihypertensive regimen and includes principles of healthy lifestyle (regular exercise, low sodium diet, limiting alcohol intake, etc.). Over 90% of our patients monitor and log home blood pressure, which facilitates medication adjustment based on home readings. A significant number of patients have a white coat hypertension, so the use of clinic readings underestimates true blood pressure control [28,70]. With the implementation of and increased patient engagement in the EHR, it may become possible to report performance based on home readings. Future initiatives for blood pressure control include having patients enter their home readings into the MyChart portal of the EPIC EHR using their mobile device or laptop so values are recorded and actionable. We have not yet implemented this blood pressure initiative due to a lack of educational materials and resources to educate patients on this electronic reporting.

### 4.2. Education

Education on CKD is critical in empowering patients to be active participants in their care and has been associated with decrease in hospitalizations and mortality [1,3,4,5,7,8,11]. In a prospective, randomized, controlled trial of an IP CKD educational intervention, the IP care group showed a significant delay in initiation of dialysis therapy compared to the usual care group (*p* < 0.0001) [78]. Pre-dialysis education is important in assuring a smooth transition to dialysis including placement of permanent access and/or transition to transplantation [79]. Peritoneal dialysis (PD) and home hemodialysis (HD) are underutilized in the United States with ~90% of patients receiving in-center HD [80]. We believe that extensive education provided to our patients was the reason for a relatively high percent of our patients starting renal replacement therapy with PD (30%) compared to in-center HD (70%). This is consistent with other studies that showed that CKD education is associated with increased selection of home HD and PD modalities as opposed to in center HD [81]. In a survey of practicing nephrologists, over 90% of the nephrologists would choose home dialysis for themselves, yet few CKD patients are on home dialysis therapy [82]. Clinicians should apply the same standards for taking care of patients that they would desire for themselves or family members, should they develop ESRD. Various medical programs are increasingly adopting technology solutions to support self-management practices [83,84]. We were able to educate many more patients with online videos than group classes (70% vs. 33% respectively). Online education provides the solution to several barriers faced with in-person education including transportation to the facility, scheduling, learning pace (i.e., patients can watch videos at their convenience and pace), and frequent physician visits or hospitalization. One major limitation is the production of videos in different languages. Due to limited resources, we did not translate the education videos into Spanish; consequently, not every patient benefited from the videos. 

### 4.3. Vascular Access

Timely permanent access creation for chronic dialysis is complicated by numerous clinical and psychosocial factors making this an important but challenging quality metric. Use of AVF for HD is associated with improved mortality and morbidity and lower cost compared to the use of a central venous catheter [10,72,85]. Over the last decade, the rate of AVF use in prevalent dialysis patients has improved significantly from approximately 35% to 65% [80]. However, at dialysis initiation, AVF use continues to be very low with over 80% of patients initiating dialysis using a tunneled catheter [80]. Emergent start of dialysis continues to be too common and likely contributes to high mortality and morbidity in the first 6 months of starting dialysis, especially in patients over 65 years of age [80].

Early in our program development, we experienced significant delays from patient referral to vascular surgery to the actual visit and/or placement of permanent vascular access. In order to address this problem, we created a joint CKD-vascular surgery clinic, scheduled once a month, where patients who had advanced CKD could see the nephrologist and surgeon during the same visit. This coordination of care resulted in timely vascular access evaluation and surgery. On average, 77% of patients started HD with a functional AVF in the first 2-year cycle and 61% in the second cycle. One challenge we encountered with this quality indicator is the small number of patients who transition from CKD stage 5 to HD making it difficult to compare and trend month-to-month data or to demonstrate significant improvement. Our results are similar to other studies demonstrating higher AVF rates of 45.2–68.4% in patients receiving IP CKD care compared to 4.8–58.8% in the usual care groups [1,4,86,87]. Despite receiving comprehensive education and IP care, there are patients who will start HD with a tunneled catheter for multiple reasons including: (1) late referral of patients with advanced CKD and low socioeconomic status, (2) emergent dialysis for acute kidney injury in patients who previously had moderate (not advanced) CKD at baseline and (3) patients who initially choose peritoneal dialysis, yet start with HD due to unforeseen acute deterioration in health. We are developing and implementing a protocol for urgent start PD (within 24–48 h after placement of PD catheter) to address the latter problem.

### 4.4. Transplantation

Survival rates for patients with ESRD are much better for those undergoing kidney transplantation compared to those receiving chronic dialysis [80]. Our program ensures timely referral of appropriate candidates to the transplant program once the GFR approaches 20 mL/min/1.73 m^2^. Our experience is that patients from our CKD IP program have better health related outcomes (i.e., health maintenance, self-monitoring of health outcomes and medication adherence) and experience a higher likelihood of placement on the transplant waiting list (not reported). However, we have not measured and compared our referral to listing ratios to that of usual nephrology care. Some patients receive preemptive kidney transplantation while others start accruing waiting time prior to initiation of dialysis (i.e., once the GFR is below 20 mL/min/1.73 m^2^). To facilitate transplant referral, we have worked with the transplant program and informatics team to enable the clear and visible display of transplant listing status in each patient’s EHR.

### 4.5. Vaccinations

Vaccinations are one of the most beneficial health prevention strategies to reduce morbidity and mortality associated with communicable infections. Patients with CKD should receive an annual influenza vaccine, pneumococcal and hepatitis B vaccinations [71]. We focused on pneumococcal rather than hepatitis B vaccination since our baseline rate for hepatitis B vaccination was high whereas there was an opportunity for improvement in pneumococcal vaccination rate. To improve this measure, we ensured accurate documentation of vaccination history by the pharmacist for every patient and we streamlined the vaccination ordering process. We experienced a robust increase in pneumococcal vaccine administrations from a baseline of less than 50% to over 90% patients at the end of the reporting cycle.

### 4.6. Advanced Directives

During the re-certification process, there is opportunity to discuss the retirement of measures and adoption of new quality measures. Our Joint Commission surveyor felt that providers in IP CKD clinic are in a unique position to discuss transitions in care and patient preferences and suggested we start tracking discussions about advanced directives with our patients. Discussion of advanced directives can be uncomfortable especially in an ambulatory care setting and with younger patients. Our social worker felt best prepared and positioned to lead the discussions with patients. Despite our perceived concerns around this measure, we were able to initiate conversations about advanced directives in 90% patients, which was a significant improvement from a baseline value of less than 30%. In 2015, Medicare spending exceeded $64 billion for beneficiaries with CKD and $34 billion for ESRD costs totaling over $98 billion [80]. The cost is disproportionally high for dialysis patients in the last year of their life. Discussing advanced directives with pre-dialysis and dialysis patients is critical to select medical interventions that are aligned with patient preferences, while reducing unnecessary cost to society.

### 4.7. Cardiovascular Disease Risk

Cardiovascular disease (CVD) remains the leading cause of death in the United States and most other developed countries [88]. Among patients with CKD, death from CVD is far more common than progression to ESRD. CKD has been identified as an independent risk factor for CVD, even after adjustment for usual comorbid conditions [74]. The risk for CVD increases as GFR declines [80,89]. Assessing the risk is critical given high prevalence and poorer prognosis after a CV event in patients with CKD compared to general population (i.e., adjusted two-year survival of patients with acute myocardial infarction is 81% in general population, compared to 56% for CKD Stage 4–5 [80]. Estimating risk for CVD in patients with CKD is complicated due to the presence of traditional and non-traditional cardiac risk factors. Online and smart phone risk calculators have been developed by the American College of Cardiology, available: http://tools.acc.org/ASCVD-Risk-Estimator-Plus/#!/calculate/estimate/ (Last Accessed: 6/25/18). We worked with the informatics team to implement an electronic ASCVD risk calculator in the EHR, resulting in a quick and steep increase in documentation of this important risk from zero to 100% by the end of the reporting cycle. Patients with high ASCVD risk receive additional attention in terms of education on the importance of lifestyle modifications, appropriate medical management and referrals to cardiology.

### 4.8. Other Indicators

For other indicators, we were successful in improving the rate of completion of office visit documentation in the EHR within 48 h of the office visit from below 50% to approximately 100%. We ensured that over 90% of diabetic patients had HgA1C checked at least every 6 months. We did not make much improvement in some process measures like clinic cancellation rate or access to care due to numerous factors outside of our control.

### 4.9. Challenges

Upfront cost of an IP team is the biggest challenge to implementing and maintaining IP CKD care. The standard fee-for-service model in the United States does not reimburse many team members other than physicians or advanced practice professionals (physician extenders). Dieticians are reimbursed by Medicare for evaluating patients with CKD stage 4, though we encountered logistical challenges with scheduling patients for two separate visits (with the physician and dietician) during the same CKD visit, obtaining insurance authorization for each visit with the dietitian and placing a physician referral to the dietician for each subsequent visit. Medicare provides reimbursement for 6 educational sessions on dialysis modalities, but these sessions must be of at least 30 minutes duration and provided by a physician or physician extender. Medication therapy management services provided by the pharmacist are not reimbursable under the current Medicare structure since the CKD visit is done in conjunction with the physician. Social worker services are not reimbursable unless counseling is provided for a mental health condition. However, IP CKD programs have a potential for creating downstream or indirect revenues by increase in outpatients starts of dialysis, more patients starting dialysis with a permanent access, more PD utilization, improved referrals for living kidney donor transplantation and in turn higher rates of transplantation. All of those help to offset the cost of IP care or even make IP CKD programs cost effective.

In 2015, Medicare ESRD expenditure/person/year was $88,750 for a HD patient, $75,140 for a PD patient and $34,084 for a transplant patient [80]. Patients who receive dialysis with AVF have a lower total cost/member/year compared to those with HD catheters [80]. More recently, Lin and colleagues evaluated the cost-effectiveness of a theoretical IP CKD program compared to usual CKD care in U.S. Medicare beneficiaries with stage 3 and 4 CKD between the age of 45 and 84 years. The results of the model showed that a Medicare-funded IP CKD program could be cost effective by decreasing the need for RRT and prolonging life [13].

Space may also pose a challenge when developing a new program. We initially secured space by sharing the same clinic space and time allocation as the general nephrology clinics. This was not as challenging compared to securing new space as part of our goals to expand the program. We struggle with obtaining new space to add additional clinics in other geographic locations to better serve our diverse patient population.

### 4.10. Limitations of Our Study

There are several limitations to our study. This is an observational, descriptive study evaluating the impact of Joint Commission certification and recertification on clinical and process of care outcomes for patients with CKD. Since there was no control group in this study, it is possible that clinical outcomes achieved in this program could be achieved in a physician-based clinic. However, various team members led many of the quality improvement projects (i.e., vaccination rates by pharmacist, advanced directives by social worker, permanent dialysis access by a nephrologist) and it is unlikely that one team member alone could do all of it. This shared approach was critical to our success and this degree of quality improvement would not likely be sustained over a long period of time in a physician-based clinic. Secondly, some of the patients referred to our program received care in the general nephrology clinic prior to referral and it is possible the previous care had impacted their outcomes, but this bias may have been in both directions for the outcomes of interest. Lastly, the results may not be generalizable to non-academic programs given differences in the informatics resources, patient population and IP team members. Additional research is needed to compare the outcomes and cost-effectiveness of IP care to usual CKD care and to evaluate the feasibility of disseminating this model of care to other institutions.

## 5. Conclusions

Joint Commission certification requires the development and implementation of robust quality assurance and performance improvement plans. CKD care delivery involves a set complex processes and improvement in outcome measures are best achieved using a team-based approach where high quality care is a priority for all IP team members. Achieving certification is not a simple task, it requires strong leadership, dedication, time commitment and institutional support with the reward of nationally recognized, external validation of the excellence in care to the patients we serve.

## Figures and Tables

**Table 1:** Summary of performance measures.

Indicators	Type/Definition	Target Goal	Rationale	Time Period of Implementation
Systolic and diastolic blood pressure	Clinical Median SBP and DBP values.	SBP < 130 DBP < 80	The control of blood pressure in the United States continues to be suboptimal. Among adults with hypertension, 48% were at goal [70]. Control of blood pressure is associated with a reduction in cardiovascular morbidity and mortality and slower CKD progression.	2009–2011
BP Control	Clinical Percentage of office visits with systolic blood pressure at goal according to national guidelines.	Positive trend		2011–2015
Hemoglobin	Clinical Median hemoglobin value.	10.5–12 g/dL	The target hemoglobin in CKD is controversial [17,38]. Studies have demonstrated that normalizing the hemoglobin value with erythropoietin stimulating agents results in increased risk of cardiovascular morbidity and mortality.	2009–2011
Pneumococcal vaccinations	Clinical/Percentage of patients with documented vaccination with Prevnar 13^®^ and Pneumovax 23^®^.	Positive trend	Patients with CKD are at increased risk of pneumococcal infection and vaccination is recommended by the Centers for Disease Prevention and Control [71].	2011–2013
Fistula at time of dialysis initiation	Clinical/Percentage of patients starting hemodialysis with arteriovenous fistula (AVF) in place.	Positive trend	AVF use for hemodialysis is associated with improved morbidity and mortality and lower costs compared to the use of a central venous catheter. Despite this, use of CVC nearly exceeds 80% in patients initiating hemodialysis. In 2006, the Centers for Medicare and Medicaid set a 66% national prevalent AVF goal, resulting in improvements in prevalent but not incident hemodialysis patients [72,73].	2013–2017
Vascular access and kidney transplant referral	Process of Care/Percentage of medically appropriate patients with eGFR < 20 mL/min/1.73 m^2^ with referral to vascular access and/or transplantation. Not all patients are transplant candidates and we use criteria from the transplant program to screen for referral (i.e., age less than 70 years, no active cancer in the past 5 years and adherent to therapies). Patients who decline dialysis and/or chose palliative care are not referred to vascular surgery.	Positive trend	Standardizing the referral process for vascular access and transplantation using specific criteria would improve rates of timely and appropriate referrals.	2009–2011
Advanced Directives	Process of Care/Percentage of patients with whom advanced directives were discussed.	Positive trend	Nephrologists caring for CKD patients are in a position to discuss transitions in care and patient preferences.	2011–2013
Patient Education	Process of Care/All new patients receiving education on CKD within 3 months of entering the program.	Positive trend	Patient education can increase knowledge of CKD progression and complications with the goal of increasing patient engagement.	2009–2011
	Process of Care/Online education viewing.			2015–2017
Testing of Hemoglobin A1c	Process of Care/All patients with DM and CKD stage 2–5 with HgA1c tested in last 6 months.	90%	Tight control of glucose is associated with a reduction of microvascular and macrovascular complications. Patients with controlled diabetes should have HgA1c checked every 6 months and if uncontrolled every 3 months [61].	2015–2017
Access to care	Process of Care/Median days to first appointment.	Negative trend	Two half day clinics limits the number of visits. Patients experienced long waiting periods from referral to first appointment.	2013–2015
ASCVD risk estimation	Process of Care/Percentage of patient visits with ASCVD risk estimated and documented.	Positive trend	Cardiovascular disease is the leading cause of death in patients with CKD. The ASCVD risk calculator provides an estimate of a patient’s risk for a cardiovascular event with the goal of reducing the risk with medical management and lifestyle modification [74].	2015–2017
Cancellation rate	Financial/Percentage of office visits cancelled by patients.	Negative trend	Patients with CKD have numerous barriers to their access to care. Evaluating the clinic cancellation rate and reasons may improve the appointment process and access to CKD care.	2011–2013
Encounter documentation	Financial/Percentage of office visit encounters with complete documentation within 48 h.	Positive trend	Complete encounter documentation is required to effectively bill for services.	2013–2015

BP = blood pressure, SBP = systolic blood pressure, DBP = diastolic blood pressure, CKD = chronic kidney disease, AVF = arteriovenous fistula, CVC = central venous catheter, eGFR = estimated glomerular filtration rate, DM = diabetes mellitus, ASCVD = atherosclerotic cardiovascular disease.

**Table 2:** Patient demographics.

Characteristic	n = 440
Age, years (mean ± SD)	64.2 ± 14.5
Gender, Male (%)	55
Ethnicity, Hispanic (%)	24
CKD Stage (%)	
1–2	8
3	51
4	24
5	17
Urine protein to creatinine ratio, mg/mg (median, range)	325 (0–31,552)
Co-morbidities (%)	
Diabetes	50
*Hypertension*	92

**Table 3:** Performance measurement report: 2009–2011.

	Reporting Year 1				Reporting Year 2			
Performance Indicators	Jul-Sep	Oct-Dec	Jan-Mar	Apr-Jun	Jul-Sep	Oct-Dec	Jan-Mar	Apr-Jun
All patients (N)	190	219	198	199	191	199	208	216
Median SBP (mmHg)	136	137	135	132	127	131	133	131
Median DBP (mmHg)	73	74	74	70	70	72	73	72
Median Hemoglobin (g/dL)	11	11	12	11	12	11	11	11
Patients with Referral to Vascular Surgery (%)	96	100	96	100	100	100	91	100
Patients with Referral to Transplant Program (%)	88	100	100	100	100	100	81	100
Patients Attending Patient Education Classes (%)	29	33	50	50	100	100	100	100

CKD = chronic kidney disease; DBP = diastolic blood pressure; SBP = systolic blood pressure.

**Table 4:** Program performance measurement report: 2011–2013.

	Reporting Year 1				Reporting Year 2			
Performance Indicators	Jul-Sep	Oct-Dec	Jan-Mar	Apr-Jun	Jul-Sep	Oct-Dec	Jan-Mar	Apr-Jun
All patients (N)	209	210	223	227	228	214	211	225
Patients with SBP ≤ 130 mmHg (%)	57	54	51	51	45	47	49	58
Patients with SBP ≤ 140 mmHg (%)	79	88	79	85	75	74	79	82
Patients with Pneumococcal Vaccine (%)	49	61	69	84	88	89	93	93
Patients with Advanced Directive Addressed (%)	29	75	94	93	94	93	93	89
Office Visit Cancellation Rate (%)	-	28	25	19	23	22	25	21

SBP = systolic blood pressure.

**Table 5:** Performance measurement report: 2013–2015.

	Reporting Year 1				Reporting Year 2			
Performance Indicators	Jul-Sep	Oct-Dec	Jan-Mar	Apr-Jun	Jul-Sep	Oct-Dec	Jan-Mar	Apr-Jun
All patients (N)	240	234	241	156	256	259	223	136
Patients w/SBP ≤ 130 mm Hg (%)	53	56	55	55	-	-	-	-
Patients w/SBP ≤ 140 mm Hg (%)	81	84	82	85	85	79	82	83
Patients w/AVF or Graft at Dialysis Start (%)	100	100	100	100	60	25	75	50
Median Days from Referral to First Appointment	17	13	7	7	9	37	12	14
Notes Closed within 48 h (%)	45	98	96	99	92	95	98	100

AVF = arteriovenous fistula, SBP = systolic blood pressure.

**Table 6:** Performance measurement report: 2015–2017.

	Reporting Year 1				Reporting Year 2			
Performance Indicators	Jul-Sep	Oct-Dec	Jan-Mar	Apr-Jun	Jul-Sep	Oct-Dec	Jan-Mar	Apr-Jun
All patients (N)	216	252	219	247	224	225	212	243
% Patients w/AVF or Graft at Dialysis Start	100	89	50	100	0	44	56	50
% Patients w/Online Patient Education	0	10	25	35	48	55	64	71
% Patients w/DM and HgA1c Order within 6 mo	90	94	90	93	89	91	97	98
% Patients w/ASCVD Risk Documentation	82	100	99	100	98	99	100	100

ASCVD=atherosclerotic cardiovascular disease; AV = arteriovenous fistula, DM = diabetes mellitus, HgA1c = glycated hemoglobin, mo = months.

## Appendix A

**Table A1:** Chronic kidney disease program team roles and responsibilities.

Medical Director	1. Medical history
	2. Physical exam
	3. Orders for encounter (lab tests, referrals, medications, follow-up)
	4. Documentation of visit in EPIC
	5. Supervises nephrology fellow and medical residents
	6. Supervises interprofessional team
	7. Plan and present classes on kidney disease
	8. Oversees medical management for CKD population
	9. Strategic planning with respect to program growth, outcomes
	10. Participate in staff evaluations
	11. Attend and direct team meetings
Pharmacist and Program Administrator	1. Medication history and medication reconciliation
	2. Medication therapy management (evaluate doses for renal function, etc.)
	3. Assist with orders for encounter (lab tests, referrals, medications, follow-up)
	4. Counsel patient on new medications, medication changes and provide a current list of their medications
	5. Documentation of visit in EPIC
	6. Supervises pharmacy residents and students
	7. Supervises interprofessional team
	8. Plan and present classes on kidney disease
	9. Responsible for analyzing and presenting program outcomes
	10. Staff recruitment and performance appraisal
	11. Lead team meetings
	12. Strategic planning with respect to program growth, outcomes
	13. Prepare budget annually
Dietitian	1. Evaluate nutritionally relevant information
	2. Assess diet and make recommendations for changes in diet or dietary supplements
	3. Document assessment, care plan and education in EPIC
	4. Plan and present classes in nutrition
	5. Create meal plans for individual needs
	6. Monitor dietary change and provide feedback
	7. Attend CKD team meetings
Case Manager	1. Brief psychosocial assessment on all new patients and document in EPIC
	2. Assess for changes on return visits
	3. Address any insurance and community resource needs with patients as appropriate
	4. For patients in CKD IV or higher, begin discussing dialysis plans, preference for PD versus HD and location
	5. Assist in teaching Modalities (Kidney Treatment Options) class to new patients and document their attendance and preference in EPIC progress notes
	6. Refer patients anticipated to need dialysis for insurance verification
	7. Assisting with transition to dialysis
	8. Assist with placement in long term facilities or communication with outside facilities
	9. Facilitate communication between patients, CKD team members and other medicine/surgical disciplines (example vascular access, interventional radiology)
	10. For any unfunded or partially funded patients; notify dialysis administrator and clinical service chief and request temporary acceptance until funding is secured
	11. Attend CKD team meetings
Nurse	1. Schedules patients into the CKD clinic
	2. Triages new referrals to CKD clinic
	3. Reviews clinic schedule every week to ensure appropriate numbers of patients
	4. Prints out the after visit summary and discharges patient from the visit
	5. Reviews next appointment, lab work needed for appointment, procedures, referrals and medication changes/prescriptions
	6. Confirms patients understanding of care plan
	7. Administers erythropoietin stimulating agents in clinic when prescribed
	8. Administers vaccinations in clinic when prescribed
	9. Documents in EPIC
	10. Receives patient calls and requests for refills from call center and triages these to appropriate individuals
	11. Attend CKD team meetings
Medical Assistant	1. Takes vital signs on patient (blood pressure, pulse height and weight)
	2. Puts the patient into the rooms
	3. Notifies CKD team of patient arrival
	4. Triages late appointments with Medical Director or Program Administrator
Patient Education Coordinator/Administrative Assistant	1. Schedule team meetings, create agendas and attend meeting
	2. Maintain SharePoint site for communications
	3. Coordinates all aspects of patient education classes (mailings, patient outreach, coordinate logistics for rooms, audio-visual, and refreshments, speakers, handouts)
	4. Maintains database of all clinic patients
	5. Prepare and mail new patient education packets
	6. Collect patient data for quality indicators database
	7. Maintain office and educational material supplies
	8. Program coordinator for 10-week Wellness Program. Responsible for brochure, mailings, patient outreach, scheduling logistics for rooms, audio-visual, and refreshments, speakers, handouts
